# Factors Associated with In-Hospital Mortality in Adult Patients with Community-Acquired *Staphylococcus aureus* Bacteremia: A Cross-Sectional Study

**DOI:** 10.30699/ijp.2025.2052150.3409

**Published:** 2025-08-15

**Authors:** Sogol Alikarami, Ghazal Razani, SeyedAhmad SeyedAlinaghi, Arash Seifi, Alireza Abdollahi, Nazanin Anaraki, Sara Ghaderkhani

**Affiliations:** 1 *Pain Research Center, Neuroscience Institute, Imam Khomeini Hospital Complex, Tehran University of Medical Sciences, Tehran, Iran*; 2 *School of Medicine, Tehran University of Medical Sciences, Tehran, Iran*; 3 *Iranian Research Center for HIV/AIDS, Iranian Institute for Reduction of High Risk Behaviors, Tehran University of Medical Sciences, Tehran, Iran*; 4 *Research Development Center, Arash Women Hospital, Tehran University of Medical Sciences, Tehran, Iran.*; 5 *Department of Infectious Diseases, Imam Khomeini Hospital Complex, Tehran University of Medical Sciences, Tehran, Iran*; 6 *Department of Pathology, Imam Hospital Complex, School of Medicine, Tehran University of Medical Sciences, Tehran, Iran*

**Keywords:** Staphylococcus aureus, bacteremia, mortality, community-acquired infections, methicillin resistance

## Abstract

**Background & Objective:**

Community-acquired *Staphylococcus aureus* bacteremia (CA-SAB) is associated with substantial morbidity, mortality, and healthcare costs. This study aimed to identify clinical and laboratory factors associated with in-hospital mortality among patients with CA-SAB.

**Methods:**

This retrospective cross-sectional study was conducted at a tertiary referral hospital in Tehran, Iran. Adult patients with positive blood cultures for *S. aureus* who met CA-SAB criteria were included. Demographic, clinical, and laboratory data were collected from medical records. The primary outcome was in-hospital mortality. Univariate and multivariate logistic regression analyses were performed to assess associations with mortality.

**Results:**

A total of 114 patients with CA-SAB were included. No significant association was observed between underlying comorbidities and mortality. Although methicillin-resistant *S. aureus* (MRSA) infection was associated with a higher mortality rate, this difference was not statistically significant (P = .32). Multivariate analysis revealed that older age (odds ratio [OR], 1.053; 95% CI, 1.012–1.095; P = .01), elevated C-reactive protein (CRP) levels (OR, 1.016; 95% CI, 1.005–1.028; P < .01), and lower serum albumin levels (OR, 0.249; 95% CI, 0.097–0.642; P < .01) were independently associated with in-hospital mortality.

**Conclusion:**

Although age was not significant in univariate analysis, it emerged as a significant predictor after adjustment for other variables. Routine laboratory parameters such as CRP and albumin may serve as valuable prognostic indicators. Early identification of high-risk patients using these markers could inform timely interventions and improve outcomes in CA-SAB.

## Introduction

*Staphylococcus aureus* (*S. aureus*) is a gram-positive bacterium commonly present in the normal flora on the skin and nasal passages. However, it can become pathogenic and disseminate into the bloodstream, causing bacteremia, which may progress to severe conditions such as sepsis (1). *Staphylococcus aureus* bacteremia (SAB) is associated with a mortality rate of 10–30% among patients across different healthcare systems (2). 

Community-acquired SAB (CA-SAB) is defined as the detection of at least one positive blood culture for *S. aureus* within the first two days of admission in the absence of any previous healthcare contact within 30 days prior to hospitalization (3). While healthcare-associated SAB (HCA-SAB) is more prevalent among individuals with chronic illnesses, recent hospitalizations, and invasive devices, CA-SAB mainly occurs in previously healthy patients, making its timely diagnosis and treatment troublesome (4, 5).

Unfortunately, the increasing prevalence of methicillin-resistant *Staphylococcus aureus* (MRSA) has reduced the effectiveness of standard empiric antimicrobial regimens (6, 7). CA-MRSA imposes an overwhelming economic burden on the healthcare system. Early detection and proper management can limit its spread and prevent progression and additional complications (8). 

Despite remarkable advancements in medical care and antibiotic therapy, the clinical outcomes of communicable diseases, including SAB, are still affected by underlying comorbidities, the primary source of infection, immune status, and antibiotic resistance (9). Furthermore, several clinical and laboratory markers have been linked to adverse outcomes, and identifying these indicators can aid in the early recognition of high-risk patients (10). 

Therefore, we conducted a cross-sectional study on patients with CA-SAB at one of the largest tertiary care hospitals in Iran. We aimed to investigate the associations between mortality and other poor outcomes with factors such as patient age, comorbidities, source of infection, early clinical status, laboratory markers, and antibiotic resistance profiles.

## Materials and Methods

### Study Design and Population

This cross-sectional study was conducted at a tertiary university hospital in Tehran, Iran. Medical records of all hospitalized patients from 2020 to 2022 were reviewed. Patients aged ≥18 years with at least one positive blood culture for *Staphylococcus aureus* within 48 hours of hospital admission, and no history of hospitalization, surgery, residence in a long-term care facility, or outpatient clinic visits within the preceding 30 days (meeting criteria for community-acquired SAB [CA-SAB]), were eligible for inclusion.

Exclusion criteria were as follows: (1) absence of clinical signs or symptoms consistent with bacteremia; (2) polymicrobial bacteremia or concurrent bloodstream infections with other pathogens; (3) a history of malignancy with recent chemotherapy, radiotherapy, or oncology clinic visits within one month before admission; (4) routine dialysis or nephrology clinic contact within one month prior to admission; (5) voluntary discharge before completion of treatment; and (6) incomplete electronic health records.

Sample size was calculated using G*Power software (version 3.1), based on a logistic regression model with an assumed odds ratio (OR) of 1.99, a significance level (α) of 0.05, and a power of 80%. Convenience sampling was employed, and all eligible patients meeting the inclusion criteria during the study period were enrolled. The final sample size matched the calculated requirement.

### Data Collection

Data were extracted from electronic health records (EHRs) by two independent reviewers using standardized paper forms. Discrepancies were resolved by discussion and review of the original patient records.

Collected data included demographic characteristics (age, sex) and comorbidities such as diabetes mellitus (DM), hypertension, moderate to severe kidney disease, heart failure (HF), liver cirrhosis, ischemic heart disease (IHD), cerebrovascular accident (CVA), malignancies, intravenous drug use (IVDU), and infections with human immunodeficiency virus (HIV), hepatitis B virus (HBV), or hepatitis C virus (HCV).

A history of diabetes or hypertension was considered positive if the patient was receiving treatment before admission. HF was defined by symptoms such as exertional dyspnea or paroxysmal nocturnal dyspnea, or symptom relief following diuretics, digitalis, or afterload-reducing medications. Moderate to severe kidney disease was defined according to the Charlson Comorbidity Index and included a creatinine level ≥3 mg/dL, clinical uremia, or a history of kidney transplantation. Malignancies included both solid tumors and hematologic cancers (e.g., leukemia, lymphoma).

Information on prior antibiotic and immunosuppressive therapies was also collected. Immunosuppressive therapy was defined as the use of ≥40 mg/day prednisolone (or equivalent) for ≥2 weeks (11) or use of other immunosuppressants (e.g., calcineurin inhibitors, antiproliferative agents, mTOR inhibitors) at immunosuppressive threshold levels (12). Prior antibiotic exposure was defined as ≥48 hours of antibiotic use within 30 days before admission, based on EHR documentation.

The presumed source of infection was identified through clinical symptoms, radiologic findings, or microbiological confirmation (e.g., positive smears or cultures from suspected sites). Initial vital signs and clinical features recorded at triage or during the first emergency department encounter were also documented.

Laboratory data were retrieved from the hospital information system (HIS), provided the samples were collected on the same day as the blood culture. These included white blood cell (WBC), neutrophil, lymphocyte, and platelet counts; hemoglobin; C-reactive protein (CRP); albumin; and venous blood gas (VBG) parameters.

Microbiological data were extracted from antibiogram reports. Antibiotic susceptibility testing was conducted using the Kirby–Bauer disc diffusion method in accordance with Clinical and Laboratory Standards Institute (CLSI) 2020 guidelines (13). Discs containing methicillin, clindamycin, erythromycin, ciprofloxacin, and trimethoprim-sulfamethoxazole (TMP–SMX) were applied to Mueller–Hinton agar plates inoculated with patient isolates. Zone diameters were measured to determine susceptibility or resistance.

Primary outcome was in-hospital mortality. Secondary outcomes included early mortality, length of hospital stay, intensive care unit (ICU) admission, and vasopressor use.

### Statistical Analysis

Statistical analyses were conducted using SPSS version 27.0 (IBM Corp., Armonk, NY). Categorical variables are reported as frequencies and percentages. Normality of continuous variables was assessed using the Kolmogorov–Smirnov and Shapiro–Wilk tests. Normally distributed variables are reported as means ± standard deviations; non-normally distributed variables are presented as medians and ranges. A natural logarithm (ln) transformation was applied to non-normally distributed variables. If normality was not achieved, nonparametric tests were used.

Categorical variables were compared using the chi-square or Fisher exact test. Continuous variables were compared using independent sample t tests or the Mann–Whitney U test, as appropriate. Univariate logistic regression was used to evaluate associations between baseline characteristics, clinical features, laboratory findings, and in-hospital mortality.

To minimize model overfitting, we adhered to the 10 events per variable (EPV) rule as suggested by Peduzzi et al (14). Variables with a P value < .10 in univariate analysis, as well as clinically relevant confounders, were entered into the multivariate logistic regression model using the "Enter" method. Odds ratios (ORs) with 95% confidence intervals (CIs) were reported. A P value < .05 was considered statistically significant.

### Definitions

The systemic inflammatory response syndrome (SIRS) score is considered positive if two or more of the four following items exist: body temperature > 38 or < 36 degrees Celsius, respiratory rate > 20 breaths/minute or partial pressure of CO2 < 32 mmHg, heart rate > 90 beats/minute, and WBC count > 12000 or < 4000/μL or > 10% band cells (15). 

A positive quick sepsis-related organ failure assessment (qSOFA) is used to evaluate organ dysfunction and is considered positive if at least two of the following elements are present: 1) respiratory rate greater than 22/minute; 2) systolic blood pressure ≤100 mmHg; and 3) alteration in mental status (16). We considered shock status as the presence of positive SIRS and/or a positive qSOFA score. 

Leukocytosis was defined as a WBC count greater than 12000/μL, and leukopenia was defined as a WBC count less than 4000/μL. Thrombocytopenia was considered as a platelet count of less than 150,000/μL. Early mortality was defined as death within 7 days of admission.

## Results

**Fig. 1:**
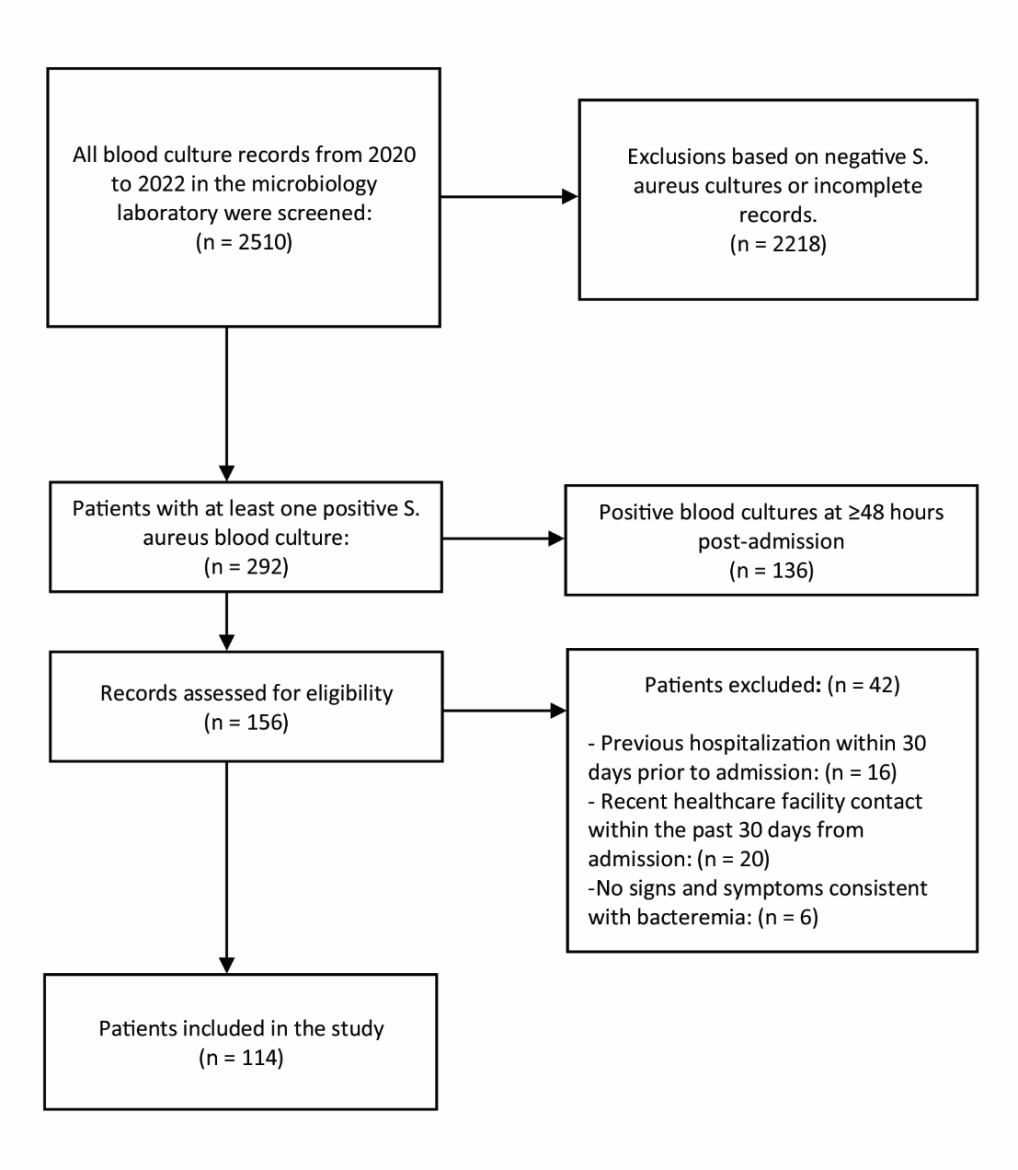
A flow diagram of participant selection

We identified 292 patients with positive cultures for *S. aureus* between 2020 and 2022. Patients whose positive cultures were obtained more than 48 hours after hospital admission (136 patients), and those who did not meet the inclusion criteria (42 patients) were excluded from the study. The final analysis was conducted on 114 participants (Figure 1).

### Descriptive data

Table 1 summarizes the demographics and clinical characteristics of the studied population. The mean age of the participants was 52.18 ± 17.60 years, and 75 patients (65.8%) were males.

DM was present in 33 patients (28.9%), and a history of hypertension was noted in 32 patients (28.1%). We included 19 patients (16.7%) with a history of malignant diseases, including 13 patients with solid tumors, four patients with lymphoma, and two patients with leukemia. None of them had received treatment at the time of admission.

Eight patients had previously received immunosuppressive therapy: two with systemic lupus erythematosus, two with rheumatoid arthritis, one with myasthenia gravis, one with a history of kidney transplantation, one with Crohn’s disease, and one with psoriasis. 

In terms of viral infections, two patients had HIV and were receiving treatment, three patients had HCV infection, but none had cirrhosis, and four patients had HBV infection, of whom two had cirrhosis.

**Table 1:** Baseline characteristics of the study population, mean ± SD or N (%).

Variable	All patients (N=114)
Age	52.18 ± 17.60
Female	39 (34.2)
DM	33 (28.9)
Hypertension	32 (28.1)
Moderate/severe kidney disease	28 (24.6)
Malignant diseases	19 (16.7)
Prior antibiotic usage	17 (14.9)
IHD	16 (14.0)
Prior immunosuppressive therapy	8 (7.0)
HF	7 (6.1)
IVDU	6 (5.3)
Liver cirrhosis	5 (4.4)
CVA	4 (3.5)
HBV	4 (3.5)
HIV	2 (1.8)
HCV	3 (2.6)

**Abbreviations:** SD = standard deviation, DM = diabetes mellitus, IHD = ischemic heart disease, HF = heart failure, IVDU = intravenous drug user, CVA = cerebrovascular accident, HBV = hepatitis B virus, HIV = human immunodeficiency virus, HCV = hepatitis C virus.

### Demographics and comorbidities

The overall in-hospital mortality rate among CA-SAB patients was 37.7%. There was no statistically significant association between the in-hospital mortality rate and patients' demographic or clinical characteristics, including age, sex, or the presence of comorbidities, such as DM, hypertension, moderate or severe kidney disease, ischemic heart disease, and malignancies. Additionally, these demographic and clinical characteristics were not significantly associated with early mortality, hospital stay, ICU admission, or vasopressor requirements (Supplementary Table 1). 

### Source of infection

The respiratory tract was the most common primary infection site, followed by the skin and soft tissue. No significant association was observed between the source of infection and in-hospital mortality. However, endocarditis was associated with an increased need for ICU admission (p < 0.001). Endocarditis and bone and joint infections were associated with prolonged hospitalization (p = 0.04 and 0.02, respectively) (Supplementary Table 2). 

### Clinical characteristics at admission

Table 2 shows the associations between clinical characteristics at admission and the in-hospital mortality rate. Our findings revealed that a positive qSOFA score at admission was significantly associated with increased in-hospital mortality (p < 0.001). In contrast, a positive SIRS score was not significantly associated with in-hospital mortality (p = 0.27). Thrombocytopenia was significantly associated with both in-hospital mortality (p = <0.001) and early mortality (p < 0.01)

Other clinical and laboratory characteristics at admission, including the time from symptom onset to antibiotic initiation, leukocytosis, leukopenia, and metabolic acidosis, were not significantly associated with mortality (Table 2 and Supplementary Table 3).

**Table 2:** Associations between clinical characteristics at admission and mortality, N (%).

Clinical characteristics at admission N = 114	Mortality N=43	P-value	E. Mortality N=21	P-value
SIRS ≥ 2	11 (35.5)	0.27	4 (12.9)	0.13^*^
qSOFA ≥ 2	10 (71.4)	<0.001	1 (7.1)	> 0.9^*^
Shock	34 (53.1)	<0.001	15 (23.4)	0.01
Days from symptom onset to antibiotic initiation (≤7d)	21 (36.8)	0.84	10 (17.5)	0.60
Leukocytosis	21 (39.6)	0.61	10 (18.9)	0.94
Leukopenia	4 (36.4)	> 0.9^*^	2 (18.2)	> 0.9^*^
Thrombocytopenia	25 (58.1)	<0.001	13 (30.2)	<0.01
Metabolic Acidosis	15 (46.9)	0.58	9 (28.1)	0.06^*^

* These p-values were calculated via Fisher’s exact test.

*Abbreviations: E. Mortality = early mortality, SIRS = systemic inflammatory response syndrome, qSOFA = quick sepsis-related organ failure assessment.*

### Regression analysis of in-hospital mortality among patients with CA-SAB

Table 3 shows the univariate and multivariate analyses for in-hospital mortality of SAB patients. 

Univariate analysis revealed that lower platelet counts (OR 0.996, 95% CI 0.992–0.999; p = 0.02) and albumin levels (OR 0.299, 95% CI 0.135–0.663; p < 0.01) were significantly associated with higher in-hospital mortality. In contrast, elevated CRP levels were positively associated with mortality (OR 1.008, 95% CI 1.000–1.015; p = 0.04). 

Age, platelet count, albumin, and CRP levels were entered into the multivariate model. Multivariate logistic regression revealed that older age (OR 1.053, 95% CI 1.012–1.095; p = 0.01), higher CRP levels (OR 1.016, 95% CI 1.005–1.028; p < 0.01), and lower albumin levels (OR 0.249, 95% CI 0.097–0.642; p < 0.01) were associated with higher in-hospital mortality (Table 3).

**Table 3:** Univariate and multivariate analyses for in-hospital mortality.

Variable	Univariate analysis OR (95% CI)	P-value	Multivariate analysis OR (95% CI)	P-value
Age	1.016 (0.994–1.039)	0.15	1.053 (1.012–1.095)	0.01
Platelet count	0.996 (0.992–0.999)	0.02	0.996 (0.991–1.002)	0.18
Albumin	0.299 (0.135–0.663)	<0.01	0.249 (0.097–0.642)	<0.01
CRP	1.008 (1.000–1.015)	0.04	1.016 (1.005–1.028)	<0.01

Abbreviations: OR = odds ratio, CI = confidence interval, CRP = C-reactive protein.

### Antibiotic sensitivity

Table 4 shows the antibiotic resistance patterns of the *S. aureus* isolates in the overall population stratified by methicillin resistance status. Overall, resistance was observed in 60.5% of the isolates to clindamycin, 44.7% to trimethoprim-sulfamethoxazole (TMP–SMX), and 72.4% to erythromycin.

When stratified by methicillin resistance, MRSA isolates presented higher resistance rates to TMP–SMX and erythromycin than MSSA isolates; however, these differences were not statistically significant.

**Table 4:** Antibiotic resistance patterns among CA-SAB patients, N (%).

P-value	Total Resistance	Resistance among MRSA (N=35)	Resistance among MSSA (N=76)	Antibiotic
0.45	66 (60.5)	20 (45.7)	46 (60.5)	Clindamycin
0.88	50 (44.7)	16 (45.7)	34 (44.7)	TMP–SMX
0.14	74 (72.4)	28 (80.0)	46 (60.5)	Erythromycin

Abbreviations: MSSA = methicillin-sensitive Staphylococcus aureus, MRSA = methicillin-resistant Staphylococcus aureus, TMP–SMX = trimethoprim-sulfamethoxazole.

Table 5 shows the associations between antibiotic resistance rates and in-hospital mortality. While mortality rates were higher among patients infected with MRSA (44.4%) than among patients infected with MSSA (34.2%), this difference was not statistically significant (p = 0.32).

No significant associations were detected between mortality and resistance to other antibiotics, including ciprofloxacin, TMP–SMX, erythromycin, or clindamycin (Table 5).

**Table 5:** Associations between antibiotic resistance and mortality, N (%).

Antibiotic	Resistance Status	Mortality	Non–mortality	P-value
Methicillin	Sensitive	26 (34.2)	50 (65.8)	0.32
	Resistant	16 (44.4)	20 (55.6)	
Ciprofloxacin	Sensitive	7 (50.0)	7 (50.0)	0.90
	Resistant	11 (47.8)	12 (52.2)	
TMP–SMX	Sensitive	24 (38.7)	38 (61.3)	0.71
	Resistant	18 (35.3)	33 (64.7)	
Erythromycin	Sensitive	12 (34.3)	23 (65.7)	0.60
	Resistant	30 (39.5)	46 (60.5)	
Clindamycin	Sensitive	16 (36.4)	28 (63.6)	0.72
	Resistant	27 (39.7)	41 (60.3)	

Abbreviations: TMP–SMX = trimethoprim-sulfamethoxazole

## Discussion

We conducted a cross-sectional study to evaluate the predictors of adverse clinical outcomes in patients with CA-SAB. We extracted data from the medical records of individuals admitted to Imam Khomeini Hospital between 2020 and 2022. We evaluated the patients’ demographics, previous comorbidities, clinical conditions at hospitalization (based on the SIRS and qSOFA criteria), and laboratory findings during hospitalization and assessed their relationships with adverse clinical outcomes, including mortality rate, length of stay, ICU admission, and vasopressor requirements. 

No significant association was found between antibiotic resistance patterns and in-hospital mortality. Although age did not show a significant association in the univariate analysis, it was a significant factor after adjustment. In addition, multivariate analysis revealed that elevated CRP levels and lower albumin levels were independently associated with higher in-hospital mortality.

A retrospective cohort study evaluated the clinical outcomes of MSSA bacteremia in diabetic and nondiabetic populations in a university hospital in Belgium (a total of 248 participants). Similar to our study, this study revealed no significant association between diabetes and 30-day mortality rates. Nevertheless, unlike our findings, this study reported an increased risk of several adverse clinical effects, including metastatic infection, prolonged hospital duration, and secondary bacteremia, in the diabetic population. Additionally, an age greater than 60 years was identified as an independent risk factor for mortality (17).

Another prospective cohort study was conducted with 303 participants at a Norwegian university hospital to evaluate one-year all-cause and infection-related mortality predictors in patients with SAB. They reported that age between 70 and 79 was an independent predictor of mortality. These findings, consistent with our own findings, suggest that advanced age may be a significant predictor of overall mortality in SAB patients (18).

A prospective multicentric cohort study was conducted in Turkey to evaluate the predictive factors for mortality in SAB patients. According to this study, methicillin resistance, ICU admission, and previous exposure to antibiotics were strong predictors of mortality. However, consistent with our study, no significant difference in terms of sex was found between the fatal and survivor groups (19).

Our study reported no significant association between underlying cirrhosis and mortality. However, several previous studies have reported increased mortality rates in SAB patients with cirrhosis (20, 21). These discrepancies emphasize the need for further research on different samples with different demographic characteristics to reach a more precise overview in the future.

Approximately 30% of the SAB patients in our study had no identifiable source. The majority of infections originated from the respiratory tract, skin, or soft tissues, and no significant associations were found between unidentified sources and adverse outcomes. However, a prospective study in nonneutropenic cancer patients with SAB reported that an undetermined source of infection was strongly associated with poor outcomes (22). 

Our study demonstrated that endocarditis as the initial source of SAB was associated with prolonged hospitalization and increased odds of being admitted to the ICU. Additionally, patients with endocarditis tended to have higher mortality rates (p-value = 0.08), which was not statistically significant in comparison to the other sources of infection. These findings contrast with other studies identifying endocarditis as a major risk factor for mortality (23, 24). 

In our study, methicillin resistance was followed by a nonsignificantly higher mortality rate (p-value = 0.32). It did not contribute to other adverse clinical outcomes, such as longer hospitalization and increased need for ICU or vasopressor therapy. These results contrast with findings from a meta-analysis that demonstrated that MRSA infections were strongly associated with increased mortality compared with MSSA infections (25). These discrepancies may reflect differences in confounder adjustment, local antimicrobial use patterns, or resistance prevalence.

We entered age, sex, comorbidities, SIRS status, qSOFA status, and laboratory markers into the univariate analysis to determine potential mortality-related factors. Variables with p-values less than 0.1 and age as potential conceptual confounding factors were included in the multivariate analysis. Our univariate analysis revealed that a lower platelet count was significantly associated with mortality. Some studies also reported a strong association between thrombocytopenia and poor clinical outcomes (26, 27). However, in the multivariable model and after adjusting for confounders, platelet count was not an independent predictor, suggesting that other variables may influence it.

Capillary leakage and increased vascular permeability are recognized as strong predictors of systemic inflammation and are, therefore, associated with worse outcomes in sepsis, including multiorgan failure (28). Albumin extravasation and lower serum albumin levels can conceptually be markers of more severe infection in SAB patients. During intense inflammation, pro-inflammatory cytokines such as IL-6 stimulate hepatocytes to prioritize the production of acute–phase proteins, such as CRP and fibrinogen, instead of albumin. These mechanisms provide a pathophysiological rationale for the associations between lower albumin and higher CRP levels with poor outcomes in SAB patients (29). However, findings from clinical practice do not always align with this theoretical framework. Contradictory results have been reported in different studies and settings. While Kim et al. and Turcato et al. reported an association between lower albumin levels and mortality (30, 31), Magnussen et al. suggested that albumin levels primarily reflect acute disease severity rather than independently predict outcomes (32).

Our findings suggest a significant prevalence of antibiotic resistance among *S. aureus* strains to commonly used antibiotics. Compared with a previous cross-sectional study conducted at the same center on 576 isolates, our findings suggest an increasing resistance trend among methicillin-sensitive isolates. (33). The differences between the two studies may arise from changes in antibiotic susceptibility over time, but the limitations of the disc diffusion method should also be considered. Although this method is simple and cost-effective, it is operator-dependent and highly affected by environmental conditions.

This study was conducted in one of the major tertiary centers in Iran and provides valuable insights into CA-SAB. Nevertheless, several limitations should be considered. This study was conducted in a single-center study, and some confounding factors should be acknowledged, including differences in management protocols, when the results are generalized to other healthcare settings. Additionally, the statistical power of the observed associations would have been greater if the study had been conducted with a larger sample size, particularly for less common clinical conditions. Furthermore, the retrospective nature of this study may have introduced bias in patient selection and data collection. Future studies should provide a more holistic evaluation of other underlying conditions affecting mortality. Further prospective and multicentric studies with larger sample sizes are needed to address these limitations.

## Conclusion

In this study, comorbidities and methicillin resistance were not significantly associated with in-hospital mortality among CA-SAB patients. Elevated CRP and reduced albumin levels were independently associated with increased in-hospital mortality. Considering these laboratory markers alongside clinical judgments can help physicians identify high-risk patients early in the course of bacteremia, optimize patient care, and improve outcomes.

## Data Availability

There is no additional data separate from available in cited references.
